# Home Quarantine Induced Health Anxiety During the Beginning of the COVID-19 Pandemic – Evidence From Iraq

**DOI:** 10.1017/dmp.2021.242

**Published:** 2021-07-26

**Authors:** Perjan Hashim Taha

**Affiliations:** College of Medicine, University of Duhok, Duhok, Kurdistan region, Iraq

**Keywords:** coronavirus disease, COVID-19, health anxiety, Iraq, pandemic, quarantine

## Abstract

**Objective:**

This study aimed to assess the compliance to voluntary home quarantine and to examine the prevalence and associated factors of health anxiety among the voluntary home quarantined population during the onset of the coronavirus disease (COVID-19) pandemic.

**Methods:**

An online survey questionnaire, including the health anxiety questionnaire, was administered to 1578 eligible adults from the general population of 19 governorates of Iraq.

**Results:**

Self-reported compliance with home confinement was reported by a majority of respondents (83%) and was followed to a larger extent by young adults (62.2%), females (53.9%), unmarried individuals (56.7%), university graduates (54.5%), unemployed individuals (48.6%), and inhabitants of the northern provinces (50.2%). Compliance was significantly correlated to the level of personal knowledge on COVID-19. The quarantined individuals experienced greater health anxieties and preoccupations and exhibited increased reassurance-seeking behavior. Higher knowledge of COVID-19 was a protective factor against health anxiety.

**Conclusions:**

A significant mental health burden is discovered among Iraqis during the quarantine period. Based on the insights gleaned from this study, psychological education and interventions should be prioritized to diminish the psychological impact of the quarantine experience, especially among the high-risk groups. Improvement in compliance to quarantine can be approached by providing better information regarding the novel infection.

## Introduction

Infectious diseases originate in 1 country and spread quickly as outbreaks, epidemics, or pandemics causing high morbidity and even high death rates.^1^ The main reason for such rapid spread is the increase in international travel and development of transportation and globalization.^2,3^ In December 2019, coronavirus disease (COVID-19) was first discovered in the city of Wuhan, China, and spread quickly.^4^ The World Health Organization declared the novel virus (officially named *severe acute respiratory syndrome coronavirus 2* [*SARS-CoV-2*]) a pandemic.^5^

Intervention methods to control the transmission of infection to susceptible individuals include voluntary isolation at home and quarantine.^6^ The word *quarantine* means preventing interactions with others, as in being confined at home. Many countries requested citizens to engage in social distancing measures and demanded those who made contact with infected patients to quarantine themselves either in their houses or at a quarantine facility.^7^ Other countries declared a complete lockdown to control the dissemination of the virus.^4^

Regardless of the effective role of home quarantine in such situations to prevent further spread of the infection, it is accompanied by certain negative consequences, including financial, emotional, and psychosocial outcomes.^8^ Different levels of depression and anxiety and a wide range of other emotions, including fear, lack of control, anger, and frustration may be expressed.^9^ As social activities and face-to-face meetings decline, it has led to feelings of loneliness and irritability, which aggravate stress, anxiety, and depression among the population.^10^ The cognitive impact of a lack of social activity may include unpredictability and suspiciousness in an individual’s health condition, which is known as health anxiety. Individuals who suffer from health anxiety, which ranges from mild to severe, are convinced that they suffer from the disease and constantly worry about it.^11^ Individuals who suffer from health anxiety exhibit dysfunctional forms of reaction to illness information and may lack protective coping strategies.^12^ They negatively interpret information and engage in increased reassurance-seeking behavior. In the case of Iraq, most of the cases were imported from Iran, and there has been a significant rise in cases as well as an increased death rate. Until March 31, 2020, 694 cases and 50 deaths were reported in the country.^13^ The Iraqi authorities banned all travel from China and Iran first, and subsequently from other infected countries. In addition, public gatherings were banned and schools and universities were closed. The public health officials encouraged voluntary home quarantine and urged lockdowns in different districts of the country on different occasions in June 2020.

The adherence of the public to the preventive measures and home quarantine is crucial in preventing spread of the virus but, to the best of our knowledge, no studies have been published on health anxiety among quarantined individuals in Iraq. This study aimed to assess the compliance to voluntary home quarantine and factors influencing it; the relationship of practicing home quarantine with health anxiety and the level of knowledge on COVID-19 was measured. The prevalence and risk factors of health anxiety among the Iraqi quarantined population during the beginning of the SARS-CoV-2 (COVID-19) pandemic were also examined.

## Methods

### Design and Sampling

A cross-sectional study was conducted in 19 provinces of Iraq. The scientific approval at the College of Medicine University of Duhok (1N-22092020) was acquired. A questionnaire was produced in the SurveyMonkey program (www.surveymonkey.com; San Mateo, CA), and the online link was sent randomly using social media platforms such as Facebook Messenger, Viber, and WhatsApp. An exponential non-discriminative snowball sample distribution technique was applied. This method was used because there are no easily available data, and it is a cost-effective method. Initially, the investigator provided 19 referrals representing the focal points of 19 Iraqi governorates who, in turn, allowed for more invitations by sending the announcement link to their contacts. The respondents were encouraged to share the link to as many individuals as possible. The participants were Iraqis who were 18 years of age or older, who possessed the mental ability to consent and respond via the online mode during the early pandemic period (March–April 2020). The data gathering period started on March 21, 2020, and lasted for 3 subsequent weeks.

### Study Instrument

The online self-reported questionnaire used multiple choice questions in which the consent form appeared in the beginning. This questionnaire was translated and retranslated back by specialists in the Arabic language. The translation of the original English version of the questionnaires into Arabic was first established by a psychiatrist and a professional translator. The back translation was carried out blindly by 2 other professional translators. The 2 transcriptions were compared for possible differences and the English-Arabic combined type of questionnaire was formulated and used. The original English text was kept to be released on the questionnaire platform for those who prefer reading items in English. After they had consented to participate, they filled in their sociodemographic details such as age, gender, marital status, educational level, work status, residence, and history of chronic respiratory diseases. Next, the respondents were asked to answer several questions related to the practice of home quarantine, the general knowledge of COVID-19, and the amount of time spent focusing on COVID-19. The items had been reviewed for validity by 7 experts: 3 psychiatrists, 2 psychologists, and 2 community medicine specialists. Each question was answered by choosing 1 option on a 4-point Likert-style scale. The last question on the survey asked individuals to compare the level of anxiety experienced regarding COVID-19, in comparison to their fear from war.

The 21 items on the health anxiety questionnaire (HAQ) were used to assess health anxiety.^14^ The reliability of the questionnaire was 0.876 (Cronbach’s alpha) for this study. Studies concluded that HAQ has appropriate discriminate validity and appeared to reflect characteristics consistent with the cognitive behavioral model of health anxiety. The HAQ was made up of 4 subscales: the subscale 1, “health worry and preoccupation”; subscale 2, “interference with life”; subscale 3, “fear of illness and death”; and the subscale 4, “reassurance-seeking behavior.” The items were rated based on the responses to the 4 options for every question (*not at all or rarely*, *sometimes*, *often*, or *most of the time*). A cutoff score of 20 was used to determine possible health anxiety.

### Data Analysis

The demographic data of the quarantined and non-quarantined participants were put forth in descriptive statistics. Iraq governorates were divided geographically into 3 groups: (1) the northern governorates including Duhok, Erbil, Halabja, Kirkuk, Nineveh, and Sulaymaniyah; (2) the middle governorates including Al Anbar, Babel, Baghdad, Diyala, Karbala, Salahuddin, and Wasit; and (3) the southern governorates including Basrah, Dhi Qar, Al-Qadisiya, Maysan, Muthanna, and Najaf. Non-parametric correlations were applied. The mean and the standard deviation of health anxiety levels were computed. The comparison of the total mean score of health anxiety and its subscales between quarantined and non-quarantined population was analyzed by an independent t-test. The comparison of prevalence of the health anxiety was examined using the Pearson chi-square test, and its risk factors were examined through a binary logistic regression. A *P*-value of t < 0.05 was considered significant. The statistical calculations were performed by statistical package for social sciences version 25 (IBM SPSS Statistics for Windows, Version 25.0; IBM Corp, Armonk, NY).

## Results

A total of 2200 persons visited the online questionnaire and 1578 persons completed the online forms (response rate: 1578/2200*100 = 71.7%). Of those who completed the questionnaire, 1310 (83%) practiced home quarantine. Younger individuals between the ages of 18 and 34 were more compliant to home quarantine than other age groups. Females (53.9%), unmarried individuals (56.7%), those with university degrees (54.5%), and unemployed individuals (48.6%) were more compliant with quarantine measures. Half of the individuals in the northern Iraqi provinces practiced home quarantine often or most of the time (Table 1).

**Table 1.** Sociodemographic characters of respondents participating home quarantine versus non-home quarantined (N = 1578)

	Home Quarantine			
	None or Sometimes N (%)	Often or Mostly N (%)	X^2^	*P*
Age				
18–34 years	132 (49.3)	849 (62.2)	22.890	< 0.001
35–54 years	112 (41.8)	380 (29)		
≥55 years	24 (9)	81 (6.2)		
Gender				
Male	159 (59.3)	603 (46.1)	15.669	< 0.001
Female	109 (40.7)	706 (53.9)		
Marital status				
Unmarried	119 (44.4)	743 (56.7)	13.612	< 0.001
Married	149 (55.6)	567 (43.3)		
Education level				
≤12 years	49 (18.3)	222 (16.9)	14.585	0.001
University degree or some	114 (42.5)	714 (54.5)		
Higher education degree	105 (39.2)	374 (28.5)		
Employment				
Student	60 (22.4)	533 (40.7)	32.130	< 0.001
Employed	41 (15.3)	140 (10.7)		
Unemployed	167 (62.3)	637 (48.6)		
Residence				
Rural	37 (13.8)	164 (12.5)	0.331	0.565
Urban	231 (86.2)	1146 (87.5)		
Iraqi districts				
Northern governorates	123 (45.9)	658 (50.2)	20.339	< 0.001
Middle governorates	71 (26.5)	200 (15.3)		
Southern governorates	74 (27.5)	452 (34.5)		
Chronic respiratory disease history				
No	243 (90.7)	1212 (92.5)	1.057	0.304
Yes	25 (9.3)	98 (7.5)		
Total	268 (17)	1310 (83)		

The proportion of individuals practicing home quarantine was significantly correlated to the level of general knowledge of COVID-19, although the correlation was weak (r = 0.093, *P* < 0.001). The likelihood of an individual to practice home quarantine was neither associated with the time spent by individuals in their effort to seek information on COVID-19, nor was it associated with the level of anxiety in the individual as a result of COVID-19. However, it was correlated to the level of total health anxiety scores (r = 0.081, *P* < 0.05) (Table 2).

**Table 2.** Correlations between practicing home quarantine, knowledge, time spent on, fear, and anxiety of COVID-19 (N = 1578)

	1	2	3	4	5
Practicing home quarantine	1				
Knowledge on COVID-19	0.093**	1			
Time focusing on COVID-19	0.031	0.092**	1		
Scaring of COVID-19 compared to war	0.029	0.033	0.207**	1	
Total HAQ score	0.081*	-0.1**	0.274**	0.19**	1

*Notes*: *P value < 0.05 is significant; **P value < 0.001.

This study revealed that the means of COVID-19-induced health anxiety total score and 2 of its subscales (factor 1, health worry and preoccupation; and factor 4, reassurance-seeking behavior) were significantly higher among the quarantined group (Table 3).

**Table 3.** Health anxiety subscale scores among quarantined group (N =1310) in comparison to the non-quarantined group (N = 268)

	Quarantined M (SD)	Non-Quarantined M (SD)	t	*P*	95% CI
Factor 1	1.07 (0.55)	0.97 (0.52)	2.929	0.003*	0.035–0.178
Factor 2	0.59 (0.69)	0.52 (0.54)	1.678	0.094	-0.013–0.163
Factor 3	0.85 (0.68)	0.79 (0.64)	1.171	0.242	-0.035–0.141
Factor 4	1.17 (0.67)	0.97 (0.63)	4.589	< 0.001**	0.116–0.289
Total HAQ	0.97 (0.48)	0.87 (0.45)	3.301	0.001*	0.043-0.168

*Notes*: M, mean; SD, standard deviation; t, t-test; *P*, *P*-value; 95% CI, 95% confidence interval; Factor 1, health worry and preoccupation; Factor 2, interference with life; Factor 3, fear of illness and death; Factor 4, reassurance-seeking behavior; HAQ, health anxiety questionnaire.

*P value < 0.05 is significant; **P value < 0.001.

Of the 1310 quarantined individuals, 661 (50.5%) individuals reported higher HAQ scores than the health anxiety disorder cutoff score. Individuals from a younger age group (55.5%) were more likely to have a higher percentage of health anxiety disorder. Similarly, women (55.7%) were more likely to have health anxiety disorder than men, and those who were unmarried (55.3%) were more likely to have higher percentages of health anxiety disorder than married individuals. Individuals with lower levels of educational attainment (55.4%) were more likely to suffer from health anxiety disorders than their higher educated counterparts, whereas those from the southern governorates (60.8%) experienced higher levels of anxiety disorder than those from other regions (*P* < 0.001). Logistic regression analysis showed that the risk factors of health anxiety disorder among those confined at home during the onset of the COVID-19 pandemic in Iraq included being female (OR = 1.422, *P* < 0.05), a resident in the south of Iraq (OR =1.745, *P* < 0.001), and having a history of chronic respiratory diseases (OR = 1.597, *P* < 0.05). It is noted that a higher knowledge level of COVID-19 is protective against health anxiety (Table 4).

**Table 4.** Prevalence and risk factors of health anxiety among Iraqi home-quarantined respondents (N = 1310)

	Health Anxiety				
	None N (%)	Yes N (%)	X^2^	OR	(95% CI)
Age					
18–34 years	378 (44.5)	471 (55.5)	24.652**	Referent	
35–54 years	221 (58.2)	159 (41.8)		0.677	0.458–1.001
≥55 years	50 (61.7)	31 (38.3)		0.599	0.333–1.077
Gender					
Male	335 (55.6)	268 (44.4)	16.382**	Referent	
Female	313 (44.3)	393 (55.7)		1.422*	1.129–1.791
Marital status					
Unmarried	332 (44.7)	411 (55.3)	16.208**	Referent	
Married	317 (55.9)	250 (44.1)		1.023	0.677–1.546
Education level					
≤ 12 years	99 (44.6)	123 (55.4)	18.217**	Referent	
University education	330 (46.2)	384 (53.8)		0.98	0.709–1.354
Higher degree	220 (58.8)	154 (41.2)		0.882	0.566–1.375
Employment					
Student	232 (43.5)	301 (56.5)	13.05*	Referent	
Employed	74 (52.9)	66 (47.1)		1.080	0.68–1.717
Unemployed	343 (53.8)	294 (46.2)		1.055	0.752–1.478
Iraqi districts					
Northern governorates	362 (55)	296 (45)	29.76**	Referent	
Middle governorates	110 (55)	90 (45)		1.112	0.794–1.558
Southern governorates	177 (39.2)	275 (60.8)		1.745**	1.355–2.247
Chronic respiratory disease history					
No	614 (50.7)	598 (49.3)	8.102*	Referent	
Yes	35 (35.7)	63 (64.3)		1.597*	1.023–2.493
Knowledge on COVID-19					
None or some	97 (39.9)	146 (60.1)	11.691*	Referent	
Very aware	551 (52.1)	507 (47.9)		0.669*	0.498–0.898
Residence					
Rural	82 (50)	82 (50)	0.016		
Urban	567 (49.5)	579 (50.5)			
Total	649 (49.5)	661 (50.5)			

*Notes*: *P value < 0.05 is significant; **P value < 0.001.

## Discussion

The main purpose of this study was to assess home confinement and to examine the health anxiety symptoms among home quarantined Iraqi individuals during the onset of COVID-19. The results of this study indicate that most of the Iraqi people practiced home quarantine at the onset of the spread of the virus. The study detected that the common groups compliant with home quarantine were younger adults, females, unmarried individuals, unemployed individuals, individuals with a university degree, and those residing in the northern governorates. More than half of the quarantined individuals exhibited a health anxiety disorder. Among the subscales of health anxiety, reassurance-seeking behavior was recorded to have the highest mean scores. High rates of health anxiety were found among younger individuals, females, the unmarried, lower educational attainment, and southern governorates’ inhabitants. The predictors of health anxiety include gender, the residential governate, the knowledge of the novel virus, and the incidence of chronic respiratory diseases.

During the pandemic, Iraq reported its first confirmed case of COVID-19 on February 22 in Najaf.^13^ Over the next month, the number of cases exceeded 100 in Baghdad and other Iraqi cities.^15^ At the time of conducting this study, the spread of COVID-19 was in the initial stages and the health authorities had not announced a public emergency. However, some measures were taken by the Iraqi Government. On March 23, 2020, they prohibited travel to Bahrain, China, France, Iran, Italy, Japan, Kuwait, Nigeria, Singapore, South Korea, Spain, Turkey, and Thailand and also prohibited individuals from these countries from visiting Iraq. Iraqi nationals and residents who had visited these countries in the past 14 days were asked to self-quarantine for 14 days on arrival.^13^ In addition, all those who had traveled from the above countries were required to undertake a medical examination at the border crossing. The public health officials also encouraged home confinement for all arrivals.

At that time, 83% of the study sample practiced home quarantine often or most of the time without the need for force. The high proportion of self-quarantine was observed, despite the collective nature of Iraqi culture and the dependence on socialization. The rest of the population who chose not to quarantine, despite encouragement from the government, was due to a lack of seriousness about the disease and a decision to risk contracting the disease rather than suffer the economic costs associated with a lockdown.^16,17^ This high compliance was in line with other countries. For example, in Canada, during the SARS outbreak in 2003, people also showed high compliance with quarantine, and in a telephone survey, about 97% of them agreed to quarantine if they were exposed to SARS.^18^

This study indicates that more younger adults quarantined themselves compared to those individuals in the 35–54 and ≥ 50 years age brackets. This group is more skilled in using new technology and Internet services and can enjoy these services in the comfort of their homes. Because most of the females in our sample were housewives and unemployed, they were more easily confined and could cope with being inside their homes. Therefore, they complied more easily to quarantine measures.

Our findings revealed that a better knowledge of COVID-19 is associated with more compliance with the home quarantine procedure and lesser anxiety rates. Transparent and clear public information is mandatory to minimize the panic and increase the adherence to the public health measures, including complying with quarantine restrictions.^19^ The respondents holding university or higher degrees were more likely to adhere to the quarantine as they had more awareness and a better knowledge of the novel infection. Compliance was higher when respondents understood the rationale for imposing the quarantine, which can be enhanced through improved knowledge about the relevant disease.^18^

Isolation and self-quarantine help decrease the spread of the infection, and reduce the spread among friends, relatives, and other social networks. This loneliness contributes to a drop-off in the social support gained, which, in turn, exacerbates the mental health impact of the isolation and quarantine, including an increase in the incidence of anxiety and depression of various types.^20^ In addition, long-term quarantine may aggravate anxiety, sleep problems, frustration, irritability, and the fear of contracting the virus.^19^ Misinformation and the sharing of fake news on social media also induce anxiety, stigma, and a loss of hope.^21^ The loneliness and isolation induced by home quarantine may promote an excessive use of the Internet, including for medical advice that may increase the people’s vulnerability to health anxiety.^22^

It is notable that among the quarantined population in Iraq, more than half were considered to experience health anxiety. This higher rate may be due to the ambiguity and a lack of access to information on COVID-19, in addition to reduced social support and a reduction of collective living impacted by home quarantine. Additionally, during the quarantine, one is mostly undergoing unpleasant feelings as a result of detachment from other people and a doubtfulness about the outbreak status, leading to striking effects.^7^ The recorded prevalence of health anxiety was even higher than the prevalence reported in medical clinics (14.9%–19.9%).^22^ Furthermore, the figure was higher than the anxiety rate measured on the Beck Anxiety Inventory among Chinese people during the lockdown period (29%).^23^ These differences could be explained by the tragic history of Iraq over the last few decades, past exposure to mass traumatic events, and internal and external conflicts that have overburdened the Iraqis and increased their vulnerability to psychological problems.

Among all of the subscales of health anxiety, quarantined respondents showed higher rates of “reassurance-seeking behavior.” This result is in line with the explanation provided by cognitive-behavioral theory, which explains the behavioral responses of health anxious patients.^12^ Staying indoors, interacting less with people, might put the person in a bad mood in addition to the concern about contracting the coronavirus can emphasize the reassurance-seeking behavior.

This study indicates that health anxiety was more prevalent among younger individuals who practiced home quarantine to a larger extent than individuals among other age groups. The younger individuals are more vulnerable to stress and emotional instability as they are more inclined to follow up on the news on social media than their older counterparts.^24^ Although a study in China revealed similar findings,^23^ a study in the United States found that social isolation puts older people at a higher risk for anxiety.^25^

The present findings point out that health anxiety among quarantined people was more common among females. This in in line with studies that indicated that being a female is a risk factor for anxiety and, in turn, health anxiety.^26^ In comparison to married people, those who were single may have received lesser help and support from a partner, and the lack of relationship interaction, as well as feelings of loneliness may have contributed to the higher rates of health anxiety.^27^ In contrast to the study conducted by Chen et al., lower educational levels were associated with higher rates of health anxiety.^28^ However, the fact that the sample taken consisted of purely medical staff may have contributed to the differing results.

This study also showed significant differences in the rate of health anxiety between inhabitants of different Iraqi districts. The residents from southern governorates showed higher rates of health anxiety compared to residents of other parts of Iraq. This may have been because the first cases of COVID-19 appeared in Najaf, which is located in the south of Iraq, in addition to the rapid increase of cases in this geographical area at the time of conducting this study.

The findings of this study confirmed that health anxiety is more prevalent among Iraqi’s quarantined people who have a history of chronic respiratory diseases. This is explained by a reaction to the stress of physical illness and is supported by other studies that indicate that health anxiety is higher among those with physical diseases.^29^

This study proved that the Iraqi people responded well to the voluntary home quarantine at the beginning of the pandemic. The public health policy should be better crafted to help prevent the spread of the disease by concentrating on the non-compliant groups. As the adherence to quarantine measures was positively related to the level of knowledge on COVID-19, it is recommended that providing better information on the novel disease can have a positive impact on the compliance with preventive measures. The quarantined individuals exhibited heightened anxiety and worries about their health. This turns to the mental health impact of the novel disease and addresses the needs to the awareness programs and psychological interventions.

This study has some limitations. First, because the study gathered data online rather than during face-to face interviews, the risk of exaggeration was possible. Second, because of the snowball sampling technique, it cannot be considered a representative sample. Third, it is difficult to recognize that the scores of health anxiety existed before the home quarantine. Last, this study examined only some variables to be risk factors of health anxiety and excluded many other possible predictors.

## Conclusions

In the beginning phase of the COVID-19 outbreak, Iraqi people responded well to protective measures including home quarantine. Home quarantine was followed to a larger extent by young adults, females, unmarried individuals, those with university degrees, unemployed individuals, and those living in the north of Iraq. The compliance to quarantine orders was related to the level of knowledge on COVID-19. This describes how the educational and public health fields are intimately related that interventions should depend upon. The quarantined individuals were highly concerned about their health and exhibited heightened reassurance-seeking behavior. It is recommended that the public health officials update the community and leaderships with enough data on risks and interferences. Additionally, the government should clarify the meaning of quarantine (the asymptomatic) versus isolation (the symptomatic). Furthermore, psychological education and interventions are necessary to diminish the psychological impact of the quarantine experience, which can be done by preventive medicine, universities, mass media, and through active stakeholders in the community.

## Supplementary material

For supplementary material accompanying this paper visit https://doi.org/10.1017/dmp.2021.242. click here to view supplementary material

## References

[1] Murthy S , Christian MD. Infectious diseases following disasters. Disaster Med Public Health Prep. 2010;4(3):232-238. https://doi:10.1001/dmp.2010.hcn10005. 21149220 10.1001/dmp.2010.hcn10005

[2] Dallas CR , Harris CH , Dallas CE. The potential impact of border security upon prevalence of infectious disease. Disaster Med Public Health Prep. 2018;12(5):554-562. https://doi:10.1017/dmp.2017.118. 29540245 10.1017/dmp.2017.118

[3] Xu F , Connell McCluskey C , Cressman R. Spatial spread of an epidemic through public transportation systems with a hub. Math Biosci. 2013;246(1):164-175. https://doi:10.1016/j.mbs.2013.08.014. 24018293 10.1016/j.mbs.2013.08.014 PMC7094585

[4] Sohrabi C , Alsafi Z , O’Neill N , et al. World Health Organization declares global emergency: a review of the 2019 novel coronavirus (COVID-19) [published correction appears in *Int J Surg.* 2020;77:217]. Int J Surg. 2020;76:71-76. https://doi:10.1016/j.ijsu.2020.02.034. 10.1016/j.ijsu.2020.02.034 PMC7105032 32112977

[5] Cucinotta D , Vanelli M. WHO declares COVID-19 a pandemic. Acta Biomed. 2020;91(1):157-160. https://doi:10.23750/abm.v91i1.9397. 32191675 10.23750/abm.v91i1.9397 PMC7569573

[6] Fong MW , Gao H , Wong JY , et al. Nonpharmaceutical measures for pandemic influenza in nonhealthcare settings – social distancing measures. Emerg Infect Dis. 2020;26(5):976-984. https://doi:10.3201/eid2605.190995. 32027585 10.3201/eid2605.190995 PMC7181908

[7] Wilder-Smith A , Freedman DO. Isolation, quarantine, social distancing and community containment: pivotal role for old-style public health measures in the novel coronavirus (2019-nCoV) outbreak. J Travel Med. 2020;27(2):taaa020. https://doi:10.1093/jtm/taaa020. 32052841 10.1093/jtm/taaa020 PMC7107565

[8] Viana RB , de Lira CAB. Exergames as coping strategies for anxiety disorders during the COVID-19 quarantine period. Games Health J. 2020;9(3):147-149. 10.1089/g4h.2020.0060. 32375011

[9] Canet-Juric L , Andrés ML , del Valle M , et al. A longitudinal study on the emotional impact caused by the COVID-19 pandemic quarantine on general population. Front Psychol. 2020;11:2431. https://doi:10.3389/fpsyg.2020.565688. 10.3389/fpsyg.2020.565688 PMC7531077 33071893

[10] Hwang T-J , Rabheru K , Peisah C , et al. Loneliness and social isolation during the COVID-19 pandemic. Int Psychogeriatr. 2020;32(10):1217-1220. 10.1017/S1041610220000988. 32450943 PMC7306546

[11] El-Gabalawy R , Mackenzie CS , Thibodeau MA , et al. Health anxiety disorders in older adults: conceptualizing complex conditions in late life. Clin Psychol Rev. 2013;33(8):1096-1105. 10.1016/j.cpr.2013.08.010. 24091001

[12] Hadjistavropoulos HD , Craig KD , Hadjistavropoulos T. Cognitive and behavioral responses to illness information: the role of health anxiety. Behav Res Ther. 2013;36(2):149-164. 10.1016/S0005-7967(98)00014-X. 9613022

[13] Al-Malkey MK , Al-Sammak MA. Incidence of the COVID-19 in Iraq – implications for travellers. Travel Med Infect Dis. 2020;34:101739. https://doi.org/10.1016%2Fj.tmaid.2020.101739. 10.1016/j.tmaid.2020.101739 PMC7219363 32405265

[14] Lucock MP , Morley S. The health anxiety questionnaire. Br J Health Psychol. 1996;1(2):137-150. 10.1111/j.2044-8287.1996.tb00498.x.

[15] Mikhael EM , Al-Jumaili AA. Can developing countries face novel coronavirus outbreak alone? The Iraqi situation. Public Health Pract. 2020;1:100004. https://doi.org/10.1016%2Fj.puhip.2020.100004. 10.1016/j.puhip.2020.100004 PMC7194717 34171039

[16] DiGiovanni C , Conley J , Chiu D , et al. Factors influencing compliance with quarantine in Toronto during the 2003 SARS outbreak. Biosecur Bioterror. 2005;2(4):265-272. 10.1089/bsp.2004.2.265. 15650436

[17] Rothstein MA , Talbott MK. Encouraging compliance with quarantine: a proposal to provide job security and income replacement. Am J Public Health. 2007;97(Suppl 1):S49-S56. https://doi:10.2105/AJPH.2006.097303. 17413059 10.2105/AJPH.2006.097303 PMC1854999

[18] Reynolds DL , Garay JR , Deamond SL , et al. Understanding, compliance and psychological impact of the SARS quarantine experience. Epidemiol Infect. 2008;136(7):997-1007. 10.1017/S0950268807009156. 17662167 PMC2870884

[19] Dagnino P , Anguita V , Escobar K , Cifuentes S. Psychological effects of social isolation due to quarantine in Chile: an exploratory study. Front Psychiatry. 2020;11:591142. https://doi.org/10.3389%2Ffpsyt.2020.591142. 33312141 10.3389/fpsyt.2020.591142 PMC7704437

[20] Hiremath P , Suhas Kowshik CS , Manjunath M , et al. COVID-19: impact of lock-down on mental health and tips to overcome. Asian J Psychiatr. 2020;51:102088. https://doi.org/10.1016%2Fj.ajp.2020.102088. 32302964 10.1016/j.ajp.2020.102088 PMC7151434

[21] Tang Q , Zhang K , Li Y. The important role of social media during the COVID-19 epidemic. Disaster Med Public Health Prep. 2020;epub:1-2. https://doi:10.1017/dmp.2020.330. 10.1017/dmp.2020.330 PMC7573455 32907683

[22] Tyrer P , Cooper S , Tyrer H , et al. Increase in the prevalence of health anxiety in medical clinics: possible cyberchondria. Int J Soc Psychiatry. 2019;65(7-8):566-569. 10.1177/0020764019866231. 31379243

[23] Ahmed MZ , Ahmed O , Aibao Z. Epidemic of COVID-19 in China and associated psychological problems. Asian J Psychiatr. 2020;51:102092. https://doi.org/10.1016%2Fj.ajp.2020.102092. 32315963 10.1016/j.ajp.2020.102092 PMC7194662

[24] Correa T , Hinsley AW , de Zúñiga HG. Who interacts on the Web? The intersection of users’ personality and social media use. Comput Human Behav. 2010;26(2):247-253. https://www.sciencedirect.com/science/article/pii/S0747563209001472.

[25] Bell SA , Singer D , Solway E , et al. Predictors of emergency preparedness among older adults in the United States. Disaster Med Public Health Prep. 2020;epub:1-7. 10.1017/dmp.2020.80. PMC7704536 32475374

[26] Dagher RK , Chen J , Thomas SB. Gender differences in mental health outcomes before, during, and after the Great Recession. PLoS One. 2015;10(5):e0124103. 10.1371/journal.pone.0124103. 25970634 PMC4430539

[27] Girme YU , Maniaci MR , Reis HT , et al. Does support need to be seen? Daily invisible support promotes next day relationship well-being. J Fam Psychol. 2018;32(7):882-893. 10.1037/fam0000453. 30211571 PMC6205907

[28] Chen Q , Zhang Y , Zhuang D , et al. Health anxiety in medical employees: a multicentre study. J Int Med Res. 2019;47(10):4854-4861. https://doi.org/10.1177%2F0300060519872310. 31638465 10.1177/0300060519872310 PMC6997781

[29] Tyrer P , Cooper S , Crawford M , et al. Prevalence of health anxiety problems in medical clinics. J Psychosom Res. 2011;71(6):392-394. 10.1016/j.jpsychores.2011.07.004. 22118381

