# Supplementary material for: Home Quarantine Induced Health Anxiety During the Beginning of the COVID-19 Pandemic – Evidence From Iraq
Source: Disaster Med Public Health Prep. 2021 Jul 26:1–6. doi: 10.1017/dmp.2021.242 (PMC8943275; doi:10.1017/dmp.2021.242)
Supplement: Supplementary file 1 [file S1935789321002421sup001.docx]

**Research project: COVID-19 Related Anxiety Disorder in Iraq During the Pandemic**

**Home quarantine induced health anxiety during the beginning of the COVID-19 pandemic- evidence from Iraq**

Below is a list of questions. Please carefully read each item on the list.

Select the answer that describes you, please.

**Part 1: Sociodemographic data:**

1. What is your Age (years)?

- Under 18
- 18-24
- 25-34
- 35-44
- 45-54
- 55-64
- 65+

2. What is your gender?

- Female
- Male

3. What is your marital status?

- Single
- Married
- Divorced/separated
- Widowed

4. What is your educational attainment?

- None
- Primary school
- Lower secondary school
- Upper secondary school
- Bachelor
- Master
- Doctorate

5. What is your occupation?

- Employed
- Unemployed
- Retired
- Farmer
- Student
- Health care workers
- Teacher
- Others

6. Do you live in an urban suburban, or rural area?

- Urban
- Suburban
- Rural

7. What is your governorate?

……………………………………

8. What is your Household size?

- Six people or more.
- Three to five people
- Two people
- One person

9. What is your self-rate health?

- Excellent
- Very good
- Good
- poor

10. Do you have chronic respiratory diseases?

- Yes
- No

**Part 2: Special questions on COVID 19 and your behavior:**

1. Do you practice prevention measures against covid 19 illness?

- Yes
- No

2. Do you practice home quarantine against covid 19?

- not at all or rarely
- some times
- often
- most of times

3. Do covid 19 news impaired you from sleep properly?

- not at all or rarely
- some times
- often
- most of times

4. Do covid 19 illness news impairs your eating habit?

- not at all or rarely
- some times
- often
- most of times

5. Did you think that social media news about covid 19 illness were scaring you?

- not at all or rarely
- some times
- often
- most of times

6. What time you spend to focus on the COVID-19?

- zero
- zero to one hour
- one to two hours
- more than three hours

7. Do you have Knowledge of the COVID-19?

- Extremely aware
- Very aware
- Somewhat aware
- Not so aware

8. Do you know that covid 19 is acute respiratory viral infection?

- Yes
- No

Other (please specify)……………………………

9. Which one scares you the most?

- The war is more than covid 19
- covid 19 more than war
- Both are equally

10. Do you think that you need a treatment for this anxiety?

- Strongly agree
- Agree
- Disagree
- Strongly disagree

**Part 3: Health anxiety questions.**

**Indicate how often you have been bothered in this way during the past week**

1. Do you ever worry about your health?

- not at all or rarely
- some times
- often
- most of times

2. Are you ever worried about that you may get a serious illness in the future?

- not at all or rarely
- some times
- often
- most of times

3. Does the thought of a serious illness ever scare you?

- not at all or rarely
- some times
- often
- most of times

4. When you notice an unpleasant feeling in your body, do you tend to find it difficult to think of anything else?

- not at all or rarely
- some times
- often
- most of times

5. Do you ever examine your body to find whether there is something wrong?

- not at all or rarely
- some times
- often
- most of times

6. If you have fever, cough or sore throat do you worry that it may be caused by a covid 19 illness?

- not at all or rarely
- some times
- often
- most of times

7. Do you ever find it difficult to keep worries about your health out of your mind?

- not at all or rarely
- some times
- often
- most of times

8. When you notice an unpleasant feeling in your body, do you ever worry about it?

- not at all or rarely
- some times
- often
- most of times

9. When you wake up in the morning do you find you very soon begin to worry about your health?

- not at all or rarely
- some times
- often
- most of times

10. When you hear of covid 19 illness or death of someone you know from covid 19 illness, does it ever make you more concerned about your own health?

- not at all or rarely
- some times
- often
- most of times

11. When you read or hear about covid 19 illness on TV, radio or social media, does it ever make you think you may be suffering from that illness?

- not at all or rarely
- some times
- often
- most of times

12. When you experience unpleasant feelings in your body, do you tend to ask friends or family about them?

- not at all or rarely
- some times
- often
- most of times

13. Do you tend to read up about illness and diseases to see if you may be suffering from one?

- not at all or rarely
- some times
- often
- most of times

14. Do you ever feel afraid of news that reminds you of death (such as funerals, obituary notices)?

- not at all or rarely
- some times
- often
- most of times

15. Do you ever feel afraid that you may die soon?

- not at all or rarely
- some times
- often
- most of times

16. Do you ever feel afraid that you may have cancer?

- not at all or rarely
- some times
- often
- most of times

17. Do you ever feel afraid that you might have heart disease?

- not at all or rarely
- some times
- often
- most of times

18. Do you ever feel afraid that you may have any other serious illness? such as covid 19 infection?

- not at all or rarely
- some times
- often
- most of times

19. Have your bodily symptoms stopped you from working during the past six months or so?

- not at all or rarely
- some times
- often
- most of times

20. Do your bodily symptoms stop you from concentrating on what you are doing?

- not at all or rarely
- some times
- often
- most of times

21. Do your bodily symptoms stop you from enjoying yourself?

- not at all or rarely
- some times
- often
- most of times
